# Using a social-ecological framework to examine the cognitive development of elementary school children in the U.S.

**DOI:** 10.1186/s40359-025-02416-6

**Published:** 2025-03-06

**Authors:** Hyejoon Park, Melinda McCormick, Ju Hong Park

**Affiliations:** 1 School of Social Work, Western Michigan University, 1903 W. Michigan Ave, Kalamazoo, MI 49008 USA; 2 Department of Convergence IT Engineering, Pohang University of Science and Technology, 77 Chengam-ro, Nam-gu, Pohang, Gyeongbuk, 37673 South Korea

**Keywords:** Social-ecological framework, Cognitive outcomes, Elementary school children, Community/environmental level, Family level

## Abstract

This study investigated the cognitive development of elementary school children by identifying significant factors within a social-ecological framework. By studying the association between social-ecological factors and children’s cognitive development, we identified the most significant factors that are linked to children’s cognitive outcomes. We used the 5th wave (9 years old) of the U.S. Fragile Family and Child Wellbeing Study. With a sample size of 1,722, we conducted multiple regression analyses to examine the relationship between individual, family, and school/community levels and children’s verbal skills (PPVT-III), reading comprehension (WJ-PC), digit span, and math problems (WJ-AP). We found that the school neighborhood was associated with verbal skills, math skills, and reading comprehension; mothers’ educational levels were significantly associated with verbal skills, digit span, and reading comprehension; and children’s race/ethnicity was associated with verbal skills. To enhance students’ cognitive development, policymakers and educators should prioritize the quality of schools and their learning environment. Creating a supportive and optimistic learning environment is crucial for all students, especially those dealing with obstacles such as poverty, family instability, and troubled schools. Providing parenting education may be helpful for parents with limited education levels.

## Introduction

Children’s cognitive and academic development can be influenced by various aspects of their environments. During middle childhood, a child’s social world expands significantly, with the family playing a crucial role in their lives [1]. School-aged children actively interact with their family members and the contexts to which they belong [1]. Families’ history, culture, social structure, and characteristics shape children’s emotional, social, and physical development. Nevertheless, school-aged children’s cognitive development and functions are shaped by their surrounding environment, including families, schools, and communities [1]. However, numerous studies have extensively explored the associations between cognitive or academic outcomes in early childhood, particularly within family and community contexts.

Therefore, in this study, we aimed to explore the cognitive development of school-aged children using Bronfenbrenner’s bioecological systems theory [2, 3]. In particular, Bronfenbrenner proposed that human development is influenced by the various environments or contexts in which people live and interact [4]. The relationships between individuals and their environment can impact well-being, including academic success. We adopt the elementary school children’s social ecological model to understand their influence on children’s cognitive development. During middle childhood, children’s social worlds expand to include the community and school, with the family exerting a constant influence [1]. Therefore, their experience as elementary school students would be different from that of infants and toddlers [1]. 

In order to understand school children’s developmental outcomes within extensive areas, we looked at: (a) individual-level characteristics, such as race and gender; (b) family-level characteristics, such as mother’s age, education, income, receipt of government assistance, mother’s mental health status, and marital status; and (c) community-level characteristics, such as the types of schools available within a community exploring these aspects of their social ecology using the U.S. national dataset, Fragile Family and Child Wellbeing (FFCW) Study. Our goal was to explore the impact of social-ecological environments on the cognitive development of U.S. elementary school children and to identify which specific aspects at various levels are closely linked to their cognitive growth.

## Literature review

### Social-ecological framework

The **s**ocial-ecological framework provides a theoretical model in which key factors influence participants [5]. The framework highlights that an individual’s actions are influenced by interpersonal traits, interactions, community elements, and public policies [5]. The framework also mentions that the connections between people and different environmental elements (e.g., family, school, neighborhood, and government) have a strong influence on individuals’ thoughts, feelings, and perspectives in shaping their understanding, consciousness, attitudes, and beliefs [5]. 

### Individual level

#### Race and gender.

Research has repeatedly shown over time that girls tend to outperform boys in terms of academic and cognitive outcomes, with girls averaging higher academic achievement than boys, especially if the boys come from lower socioeconomic status (SES) households [6]. In part, this may be due to differences related to self-discipline and engagement with school for girls, with more behavioral issues reported for boys, as well as a lower likelihood of completing their homework [6]. When examining race/ethnicity and gender, African American and Latinx males perform significantly poorer in school than their female counterparts [7, 8]. Students of color in the U.S. are increasing in number in public schools, but African American or Latinx students continue to lag behind White students in elementary school [8]. Some of the factors contributing to racial disparities in children’s educational outcomes include family and neighborhood poverty, parental education, and racial discrimination [3, 8–10]. Consistently, compared to the majority of poor children, children from Latino, African American, and American Indian families, as well as children from families who immigrated to the U.S., are overrepresented among all children in poverty compared to the majority of poor children [1]. 

Rasheed and colleagues [8]—studying a racially and ethnically diverse group of students and teachers in public elementary schools in New York City—found that teacher and child racial/ethnic matches were associated with improvements in engagement, motivation, social skills, and attendance and that overall classroom diversity resulted in significant improvements in reading competence. At the same time, however, Rasheed and colleagues [8] found that “even in a diverse sample, children’s cognitive outcomes still vary by teacher and child race/ethnicity” (p. 614). Additionally, Lubienski and Lubienski (2006) [11] found that “Black eighth graders scored an average of over 20% lower than white students within the same school who were identical on all other demographic measures,” (p. 683) leading them to encourage targeting inequities among students as a way to close achievement gaps in education in the U.S [11]. 

### Family level

*Mother’s Education.* Maternal education levels have been found to predict child outcomes in all academic/cognitive areas and most social variables, such that social participation and classroom skills were higher, and higher maternal education was associated with higher levels of children’s achievement [12]. Mothers’ education benefits children by providing them with various opportunities to connect with resources that can influence their environment. This includes being able to interact with their mother’s educated peers, who can act as positive role models and offer access to various social and cultural advantages [13]. Research on elementary-aged children in Portugal has shown that having parents with higher educational achievements has also been found to be associated with higher academic achievement and cognitive development for children in research on elementary-aged children in Portugal. Children of parents with higher education have greater access to educational and cultural materials than those whose parents have lower education [14]. 

*Family Structure. Single-parent household (female-headed).* Multiple risk factors impact developmental outcomes, especially poverty and single-parent-headed households. Furthermore, as children are exposed to more risk factors, the likelihood of poorer outcomes increases [12]. Fagan (2011) found that children had better outcomes for literacy if they came from families where their mothers were married, divorced/separated, single, and noncohabiting; all of these children had better literacy outcomes than those whose mothers cohabited [15]. Fagan (2011) also emphasized that children from married families may have an inherent predisposition for competence in various intercultural areas [15]. 

A study by Jagannathan et al. (2023) using the nine-year-old wave from the FFCW dataset has shown that residing in a two-parent household with strong social support can enhance academic resilience for children at this age. This research suggests that the level of family support is more crucial at age nine than at age 15, emphasizing the significance of developmental contexts for this particular age group [16]. In a separate study using the same FFCW dataset, Lee and McLanahan (2015) discovered that family instability has a more significant effect on children’s socioemotional development compared to their cognitive achievements [17]. They also observed that cognitive performance is influenced differently based on the type of change experienced; for instance, moving out of a household has a greater impact on cognition than moving into one [17]. However, transitions out of a two-parent family were found to lower children’s PPVT scores [17]. The researchers noted that the impact of family instability on cognitive achievement was more pronounced for African American children in comparison to White or Hispanic children [17]. They highlighted that these effects were less significant than the impacts of mothers with low educational levels or being raised in impoverished households [17]. Due to the varying cognitive effects based on different types of family stability, they recommended focusing on the specifics of family instability to better understand potential consequences, as their findings indicated noticeable distinctions [17]. 

*SES.* The most important variables for predicting children’s achievement were SES, social assistance, mother’s age at first birth, gender, residential mobility, the presence of ADHD/conduct disorders, and measures of family functioning (children in care, family structural issues) [18]. Higher SES and higher parental education were also found to be associated with greater academic achievement for children in the study conducted by Alves and colleagues [14]. In addition, children from lower SES backgrounds attend schools with more limited vocabulary and numeracy skills [19]. Additionally, **c**hildren born to teenage mothers are more likely to have poor outcomes and to become teen parents themselves [18]. Pilkauskas and colleagues (2018), using the longitudinal dataset from FFCW, also investigated how consistent maternal employment affects children’s cognitive abilities at ages five and nine [20]. Their study revealed that a rise in maternal employment during early childhood was linked to improved scores on PPVT and Woodcock-Johnson scores. However, these cognitive improvements were insignificant by the time the children reached age nine [20]. 

*Mother’s Depression.* An established body of research notes the relationship between maternal depression and children’s outcomes, including emotional, behavioral, and cognitive success [21–24]. Kingston and Tough (2014) found that maternal mental health problems increased the likelihood of school-aged children’s development being impacted on many levels, including cognitive development [25]. Similarly, Charrious et al. (2020) found that maternal depressive symptoms led to lower cognitive scores in early infancy [21–23]. This can result in a circular process over time in which the child’s poor cognition may affect the mother’s depression, potentially negatively impacting the child repeatedly.

### Community and environmental level

*School Type.* Education is crucial during middle childhood for developing children’s cognitive abilities. Children in this stage spend most of their waking hours at school [1]. Children entering school begin to explore a new environment that is different from the family [1]. In the U.S., there are different types of schools that students attend. Parents choose the type of school that best meets their child’s needs [26]. The common types of schools include traditional public schools, which encompass magnet schools and charter schools, as well as private schools, which consist of special education schools [27]. Traditional public schools are funded by property taxes, as well as other state and federal government funds. Students may attend their local public school for free, as determined by their place of residence [27]. These schools adhere to state-mandated rules for curriculum and governance. They offer programs for both typical students and students with special needs. Magnet schools are public schools that offer programs in STEM, technology, or the arts. These guidelines have been developed to specify study environments in the school district. To be admitted to this school, students must demonstrate their abilities in their area of specialization. The teachers at the school develop a curriculum to meet the specific objectives of magnet students [28]. 

First, with respect to inclusive settings (putting students with and without disabilities together) or segregated settings (placing students with disabilities separate from other students), Dalgaard et al. (2020) conducted a meta-analysis of publications on the benefits of inclusivity and concluded that, on average, there were no sizeable positive or negative effects seen on inclusive schooling as measured by language, literacy, and math outcomes [29]. Instead, individual assessments of student needs may be more critical in determining which learning site would be more beneficial for the student [29]. 

Second, regarding public or private schools, there has been a long-standing argument regarding whether public or private schooling provides better educational outcomes for students in the U.S. Lubienski and Lubienski (2006) studied student outcomes from grades four and eight and found that after they controlled for demographic differences for students in grade 4, public schools scored significantly higher than private schools [11]. They also discovered that charter schools performed substantially lower at the fourth-grade level compared to non-charter public schools. The authors conclude that public schools perform relatively well in comparison to private schools after controlling for student demographic characteristics. Alves and colleagues found that students in rural and public schools outperformed those in urban and private schools in Portugal [14]. 

*Neighborhood Effects*. Neighborhoods provide social networks, social capital, and other resources that have an impact beyond the characteristics of the family. Neighborhoods with more institutional resources are associated with higher grades and the presence of more positive role models [30]. Neighborhood factors play a significant role in development; neighborhood influences can impact learning opportunities and readiness [12]. 

Neighborhood characteristics such as the proportion of low-income neighbors, unemployment rate, and residential instability predicted lower academic scores and higher dropout rates [31]. Hansen et al. (2001) also found that neighborhoods with higher levels of poverty predicted lower grades in math [12]. Additionally, in these neighborhoods, hardship is negatively related to the availability of positive problem-solving solutions for children [12]. 

High-poverty neighborhoods lack opportunities, which weakens social institutions and limits access to neighbors who can provide resources [31]. At the same time, parenting and collective socialization can serve as protective mechanisms for children in these neighborhoods [31]. Additionally, neighborhood contexts and their socioeconomic and social organizational features are associated with differences in how racial identity relates to academic values and behaviors; racial pride served as a buffer to the effects of disorganized neighborhood contexts [30]. 

In a research study utilizing the fifth (nine-year-old children) and ninth waves (fifteen-year-old children) from FFCW data, Jagannathan and colleagues (2023) discovered that the academic performance of low-income children was influenced by the neighborhood they lived in [16]. They observed that low-SES children who outperformed their peers in the same state at age nine tended to reside in neighborhoods with higher levels of social cohesion and social control, as well as lower levels of poverty, violence, and racial minority composition compared to low-SES children who did not perform well academically [16]. The study also highlighted that factors such as the proportion of two-parent families in the child’s immediate area, the number of vacant houses, the poverty rate, and racial minority presence had a significant impact on the academic performance of nine-year-old children [16]. Additionally, the research indicated that academically successful low-income children were more likely to be female [16]. 

Schwartz and colleagues (2022) analyzed data from the FFCW study up to nine years and discovered that children who faced eviction during infancy, early childhood, and middle childhood had lower scores on four cognitive tests at age nine [32]. Additionally, children who experienced eviction during middle childhood had scores that were significantly lower compared to their peers with stable housing, equivalent to missing up to a year of schooling [32]. The study also revealed that eviction during infancy and middle childhood could lead to future cognitive difficulties, indicating that eviction might contribute to educational disparities based on socioeconomic status and race, potentially worsening racial inequalities in education [32]. 

Using the same dataset of FFCW, Lechuga-Peña and colleagues (2019) examined how low-income mothers received housing assistance compared to those living in public housing in terms of school-based parent involvement when their children were nine years old [33]. They discovered that housing mobility and longer work hours could hinder the ability of low-income mothers with housing subsidies to engage in school-based parent involvement, as these mothers often work extended non-traditional hours [33]. This situation resulted in less time for these mothers to provide support to their children compared to more affluent parents [33]. 

Observing the associations between significant factors within the social-ecological model and elementary school children’s outcomes suggests a necessity for a better understanding of the application of the framework to understand children’s development and assist them more effectively by suggesting salient and specific ideas of what they need for cognitive areas.

## Methods

### Data and sample

We used publicly available nine-year follow-up data (fifth wave) from the FFCW Study. The study was approved by the Western Michigan University Institutional Review Board (IRB) office (Proposal #23-0116-P00001). The data were collected from August 2007 through April 2010 and comprised interviews with core biological parents, primary caregivers, focal children, and teachers [34]. The survey questions were administered in three steps. First, the primary caregiver survey needed to be completed through computer-assisted telephone interviews along with interviews of core biological parents. Second, home visits were scheduled during the primary caregiver and core biological parent phone interviews. A 20-minute interview was conducted with the focal child using computer-assisted personal interview technology, and the primary caregiver completed a self-administered questionnaire. Finally, interviewers collected consent and contact information to mail hard copies of interviews to the focal children’s teachers [34]. All surveys and interviews were conducted in English.

Families who responded to the surveys received compensation throughout the nine-year wave of data collection. The compensation amounts were $25 for the primary caregiver, $30 for the biological mother, $75 for the biological father, $30 for the child survey, and $65 for home visit activities [35]. 

The total number of participants in the original dataset was 4,898. For our study sample selection, we first excluded 2,644 casesidentified as ‘not in the wave.’ Second, we excluded 178 cases that responded with ‘skip,’ ‘don’t know,’ or ‘refuse.’ Third, 354 cases were also deleted after using listwise deletion to treat missing values.Ultimately, this left 1,722 for the final sample used in the analysis. Overall, the analytical sample for this study consisted of 1,722 households with 9-year-old children in the U.S.

### Measures

To measure children’s cognitive abilities, four assessments were selected from the dataset.

The dependent variables included a child’s cognitive outcomes, such as PPVT-III, WJ-Applied Problems, WJ-Passage Comprehension, and Digit Span. The independent variables were the child’s social-demographic characteristics, including child’s sex and race at the individual level, mother’s age, mother’s educational level, mother’s marital status, the status of mother’s depression, household income, the status of health insurance and/or public assistance at the family level, and the school type and school neighborhood at the community level.

### Dependent variables

*PPVT-III (Verbal Intelligence).* This assessment measures receptive vocabulary and screens for verbal ability. Similar to the Woodcock-Johnson III tests, it is administered using an “easel” or activity book. The interviewer reads a word and asks the child to identify the corresponding picture from a set of four pictures [34]. 

*Digit Span (Memory Span).* This assessment evaluates a child’s auditory short-term memory, sequencing skills, attention, and concentration using the Wechsler Intelligence Scale for Children, Digit Span subtest (WISC-IV Digit Span). The In-Home Assessment contains 16 items divided into two sections from the WISC-IV Digit Span forward and backward tests. Each item contains two trials or chances for a child to repeat the span correctly. Each trial is different, but trials for each individual item are equivalent. Interviewers present a number and ask the child to repeat it, either forward or backward, depending on the section. Interviewers score 1 for correct and 0 for incorrect [34]. 

*Woodcock Johnson-Passage Comprehension (WJ-PC) (Reading Comprehension).* The initial Passage Comprehension (i.e., WJ Subset 9) items are designed to assess symbolic learning, such as matching images (e.g., matching pictography comprehension of a word with an actual picture of the object). The following items are presented in a multiple-choice format, and respondents indicate the picture represented by a phrase. The remaining items direct respondents to read a short passage and identify a missing keyword that makes sense in the context of that passage. The items become increasingly difficult by removing pictorial stimuli and increasing passage length, level of vocabulary, and the complexity of syntactic and semantic cues [34]. 

*Woodcock Johnson-Applied Problems (WJ-AP: Subset 10) (Math Problems).* This assessment requires the focal child to analyze and solve math problems. To solve the problems, the focal child must listen to the problem and recognize the procedure to be followed. The child then performs relatively simple calculations. Given the presence of extraneous information, the child must decide not only the appropriate mathematical operations to use but also which mathematical numbers for the calculation. Item difficulty increases with complex calculations [34]. 

### Independent variables

*Individual Level*. We selected the child’s sex and race/ethnicity.

*Family Level.* For this level, we selected the mother’s age, household income, mother’s marital status, the mother’s depression status, and mother’s educational level. Mother’s age and household income were continuous variables. Additionally, to determine the household’s financial condition, the question—“Are you currently covered by any type of insurance, including private insurance, Medicaid, or another public, federal, or state assistance program that pays for medical care, or do you belong to a Medicaid HMO?”— was used. The answer to this question was either “Yes” [1] or “No” (0). Mother’s marital status included being married, separated, divorced, cohabiting, romantic but not living together, and not romantic.

To obtain the condition of mother’s depression, we chose the question, “do you take prescribed medication for depression?” and the answer for it was either Yes (1) or No (0) Mother’s educational level was identified as less than high school, high school or equivalent, some college or teaching, and college or graduate degree. The final variable was obtained from the mother’s criteria for depression, as determined by mothers’ answers of either “Yes” [1] or “No” (0).

*Community/Environmental Level*. The types of schools and neighborhoods where children go were chosen based on specific school types and neighborhoods with criminal problems. There are four types of schools from the data collection: regular public school, a school with a magnet program, a school of choice, and a special education school. Concerning each school’s neighborhood type, the data inquired, “How much of a problem is a crime in the school neighborhood?” The choices for the answer were either a major problem [1], somewhat of a problem [2], or no problem [3]. These questions were answered by a focal child’s school teacher, and the answers to both questions were treated as categorical.

### Data analysis

We first estimated descriptive analyses to calculate the distributions of the studied variables (Table 1). Correlation Coefficient for binary logistic regression was used to examine the relationships among variables (Table 2). Finally, we used ordinary least squares (OLS) regressions toexamine the association between social-ecological factors and cognitive areas of elementary school-aged children (Tables 3, 4, 5 and 6). OLS is used to “explore the relationship between one continuous dependent variable and a number of independent variables or predictors,” and “it allows a more sophisticated exploration of the interrelationship among a set of variables.” [36, p.146]. This analysis helps address how well various variables predict a particular outcome [36]. Additionally, we also calculated the effect size of each model to examine the magnitude among variables. For this, we used Cohen’s *d*, which describes the standardized mean difference between two groups of independent observations for the sample as *d*_*s*_ [37].

## Results

**Table 1:** Descriptive statistics of variables (*n* = 1722)

Variables	*n*	%	Mean	SD
Independent Variables				
Sex				
Boy	884	51.3		
Girl	838	48.7		
Race/Ethnicity				
White, non-Hispanic	33	19.2		
Black, non-Hispanic	881	51.2		
Hispanic	439	25.5		
Other	72	4.2		
School type				
Public school	1494	86.8		
School with magnet programs	76	4.4		
School of choice	147	8.5		
Special education school	5	0.3		
School neighborhood with crime				
A big problem	427	24.8		
Somewhat problem	705	40.9		
No problem	590	34.3		
Mother’s Educational Level				
Less than high school	365	21.2		
High school or equivalent	379	22		
Some college or teaching	712	41.3		
College or graduate	266	15.4		
Mother’s marital status				
Married	487	28.3		
Separated	25	1.5		
Divorced	6	0.3		
Cohabiting	636	36.9		
Romantic but not living together	432	25.1		
Not romantic	136	7.9		
Government Assistance				
Yes	165	21		
No	619	79		
Mother’s depression				
Yes	301	17.5		
No	1421	82.5		
Household income			44,176.55	49,471.75
Mother’s age			34.26	6.01
Dependent Variable				
PPVT-III			92.19	18.07
WJ-AP			96.98	20.22
WJ-PC			91.57	18.58
DIGIT SPAN			9.26	3.02

### Descriptive statistics

Table 1 presents the descriptive statistics for all variables in the study. On average, the age of mothers was 34.26 (SD = 6.01). The sample’s household income, on average, was $44,176.55 (SD = $49,471.75). In terms of children’s sex, 51.3% of children were boys (*n* = 884). For the race/ethnicity distribution, 19.2% of children were White (*n* = 33), 51.2% were African American (*n* = 881), 25.5% of children were Hispanic (*n* = 439), and 4.2% of them were identified as other races (*n* = 72). For the school type, 86.8% of children were in public school (*n* = 1494), 4.4% of children were in schools with magnet programs (*n* = 76), 8.5% of children were in schools of choice (*n* = 147), and 0.3% of children were in special education schools (*n* = 5). For crime in the school neighborhood, 24.8% reported it as a major problem (*n* = 427), 40.9% reported it to be somewhat of a problem (*n* = 705), and 34.3% reported having no problems (*n* = 590). For mothers’ educational background, 21.2% had less than high school (*n* = 365), 22% had a high school or equivalent degree (*n* = 379), 41.3% had some college or teaching degree (*n* = 712), and the remaining 15.4% had a college or graduate degree (*n* = 266). For mothers’ marital status, 28.3% were married (*n* = 487), 1.5% were separated (*n* = 25), 0.3% were divorced (*n* = 6), 36.9% were cohabiting (*n* = 636), 25.1% were romantic but not living together (*n* = 432), and 7.9% were not romantic (*n* = 136). 79% reported not receiving government assistance. A total of 82.5% of mothers reported that they did not have depression. With respect to dependent variables, the mean PPVT-III score was 92.19 (SD = 18.07), the mean Digit Span score was 9.26 (SD = 3.02), the mean WJ-AP score was 96.98 (SD = 20.22), and the mean WJ-PC score was 91.57 (SD = 18.58).

**Table 2:** Bivariate analysis between variables

	(a)	(b)	(c)	(d)	(e)	(f)	(g)	(h)	(i)	(j)	(k)	(l)	(m)	(*n*)
(a) Race/Ethnicity	1													
(b)Mother’s age	-0.07 **	1												
(c)Income	-0.19 **	0.24 **	1											
(d) Mother’s marital status	0.08 **	-0.28 **	-0.39 **	1										
(e)Mother’s depression	-0.30	-0.03	-0.08 **	0.05 *	1									
(f)Mother’s education	-0.22 **	0.19 **	0.39 **	-0.21 **	-0.06 *	1								
(g)School type	-0.00	-0.02	-0.53 *	0.46	0.01	0.03	1							
(h)School neighborhood with crime	-0.18 **	0.09 **	0.29 **	-0.27 **	-0.00	0.27 **	-0.08 **	1						
(i)Public assistance	0.05 *	-0.24 **	-0.25 **	0.19 **	0.07 **	-0.16 **	0.02	-1.04 **	1					
(j)Sex	-0.03	0.01	-0.05 *	-0.01	-0.29	-0.01	0.42	-0.01	0.03	1				
(k) PPVT-III	-0.12 **	0.11 **	0.28 **	-0.17 **	-0.34	0.28 **	-0.24	0.24 **	-0.12 **	0.01	1			
(l)Digit Span	-0.09 **	0.05 *	0.16 **	-0.07 **	-0.01	0.17 **	-0.01	0.13 **	-0.06 *	0.05 *	0.45 **	1		
(m)WJ-AP	-0.08 **	0.06 *	0.23 **	-0.15 **	-0.02	0.15 **	-0.03	0.15 **	-0.10 **	0.05	0.58 **	0.49 **	1	
(n)WJ-PC	-0.11 **	0.06*	0.17 **	-0.13 **	-0.01	0.18 **	0.02	0.15 **	-0.06 *	0.10 **	0.59 **	0.48 **	0.64 **	1

Note. * *p* < 0.05, ** *p* < 0.01, *** *p* < 0.001

### Bivariate analysis

Table 2 presents the bivariate analysis of all variables in the model. The PPVT-III scores were correlated with the child’s race/ethnicity, mother’s age, household income, mother’s marital status, mother’s education level, public assistance, and school neighborhood with crime. Digit Span was correlated with the child’s race/ethnicity, mother’s age, sex, household income, mother’s marital status, mother’s depression, mother’s education level, public assistance, school neighborhood, and PPVT-III. The WJ-AP variable was correlated with the child’s race/ethnicity, household income, mother’s age, mother’s education level, mother’s marital status, school neighborhood with crime, public assistance, PPVT-III, and Digit Span. The WJ-PC variable was correlated with the child’s race/ethnicity, sex, household income, mother’s marital status and age, public assistance, mother’s education level, school neighborhood, PPVT-III, Digit Span, and WJ-AP.

After conducting univariate analysis, it was discovered that only five individuals out of the total sample of 1722 were attending a “special education school” (refer to Table 1). Subsequently, upon examining the original regression models, it was observed that this particular type of school was leading to biased and inefficient outcomes, characterized by large coefficients and standard errors due to significant overfitting issues (99.7% in 0 and 0.3% in 1). As a result, the decision was made to remove this category from the “school type” variable in the main models and to reanalyze the data to generate more reliable and efficient results. The key findings were as follows:

**Table 3:** OLS results for PPVT-III

Variables		B(SE)	b			95% CI
**Individual Level**						
Sex (boy)						
girl		1.16(1.18)	0.03			[-1.16, 3.47]
Race/Ethnicity (White)						
African American		-6.58***(2.01)	-0.19***			[-10.53, -2.63]
Hispanic		-5.31*(2.16)	-0.13*			[-9.58, -1.04]
Other		-2.16(3.69)	-0.02			[-9.43, 5.09]
		-0.12(0.12)	-0.04			[-0.35, 0.12]
**Family Level**						
Mother’s age						
Household income		4.33(0.00)	0.07			[0.00, 0.00]
Mother’s marital status (married)						
separated		-8.92(5.47)	-0.06			[-19.64, 1.81]
divorced		-13.95(9.63)	-0.05			[-32.85, 4.95]
cohabiting		-1.81(1.73)	-0.05			[-5.20, 1.59]
Romantic not living together		-3.75*(1.88)	-0.10*			[-7.44, -0.62]
Not romantic		-0.10(2.43)	-0.00			[-4.81, 4.67]
Mother’s depression (no)						
Yes		-1.56(1.48)	-0.04			[-4.45, 1.34]
Mother’s educational level (less than high school)						
High school or equivalent		1.25(1.75)	0.03			[-2.19, 4.69]
Some college teaching		4.80**(1.55)	0.14**			[1.75, 7.85]
College or graduate		9.41***(2.65)	0.14***			[4.21, 14.61]
**Community/Environmental Level**						
School type (public)						
School with a magnet program		5.84(3.26)	0.06			[-0.55, 12.23]
School of choice		1.60(2.1)	0.03			[-2.43, 5.63]
School neighborhood with crime (a big problem)						
Somewhat problem		0.73(1.44)	0.02			[-2.10, 3.56]
No problem		4.58***(1.66)	0.12***			[1.34, 7.83]
Government assistance (no)						
Yes		2.31(1.53)	0.06			[-0.70, 5.32]
Constant		109.59***(16.36)				[77.47, 141.71]

Note. OLS = ordinary least squares; reference categories are indicated in parentheses. *B* = unstandardized coefficient, SE = standard error, *b* = standardized coefficient, CI = 95% confidence interval. * *p* < 0.05, ** *p* < 0.01, *** *p* < 0.001

### Results for verbal skills

The effect size of this model is small (Cohen’s f² = 0.11)^1^. Table 3 displays the OLS regression estimates predicting PPVT-III and the other variables. In this model, we found significant relationships between children’s race and PPVT-III. African American and Hispanic children were significantly associated with lower PPVT-III scores compared to White children (*B* = − 6.58, *p* < 0.001; *B* = -5.31, *p* < 0.05, respectively). Second, children of mothers who had romantic relationships but were not living with partners had a significant relationship with lower PPVT-III scores in comparison to children whose mothers were married (*B* = -3.75, *p* < 0.05). Third, the school neighborhood was associated with PPVT-III. Children in the school neighborhood without problems showed higher PPVT-III scores than children in school neighborhoods with a big crime problem (*B* = 4.58, *p* < 0.001). Fourth, mother’s educational levels were significantly associated with PPVT-III. Children with mothers having some college or teaching and those whose mothers had completed college or graduate degrees showed higher PPVT-III than children with mothers having less than a complete high school education (*B* = 4.80, *p* < 0.01; *B* = 9.41, *p* < 0.001, respectively). In the regression model, the effect size between the predictor and dependent variables can be observed by the standardized beta, which indicates the strongest predictor (Bannon, 2019) [39]. According to the table, the strongest positive predictor of verbal skill was being the mother’s educational level (i.e., some college teaching and college or graduate level) (*b* = 0.14, *p* < 0.05; *b* = 0.14, *p* < 0.001, respectively). The strongest negative predictor of verbal skill was being African American children (*b* = -0.19, *p* < 0.001).

**Table 4:** OLS results for Woodcock-Johnson Applied problems (WJ-AP)

Variables		B(SE)	b			95% CI
**Individual Level**						
Sex (boy)						
girl		1.32(1.42)	0.03			[-1.47, 4.11]
Race/Ethnicity (White)						
African American		-3.15(2.42)	-0.08			[-7.91, 1.61]
Hispanic		-0.39(2.62)	-0.01			[-5.54, 4.75]
Other		-6.51(4.45)	-0.06			[-15.24, 2.21]
**Family Level**						
Mother’s age		-0.09(0.14)	-0.02			[-0.36, 0.20]
Household income		6.67*(0.00)	0.09*			[0.00, 0.00]
Mother’s’ marital status (married)						
separated		-2.73(6.58)	-0.02			[-15.66, 10.19]
divorced		-17.66(11.60)	-0.05			[-15.66, 10.19]
cohabiting		-2.03(2.08)	-0.05			[-6.12, 2.06]
Romantic not living together		-3.39(2.26)	-0.08			[-7.84, 1.05]
Not romantic		-0.77(2.93)	-0.01			[-6.52, 4.98]
Mother’s depression (no)						
Yes		-2.69(1.88)	-0.06			[-6.17, 0.80]
Mother’s educational level (less than high school)						
High school or equivalent		-0.78(2.11)	-0.02			[-4.92, 3.36]
Some college teaching		2.16(1.87)	0.05			[-1.52, 5.84]
College or graduate		4.88(3.19)	0.06			[-1.39, 11.14]
**Community/Environmental Level**						
School type (public)						
School with a magnet program		-3.01(3.92)	-0.01			[-6.52, 4.97]
School of choice		0.86(2.47)	0.01			[-4.00, 5.71]
School neighborhood with crime (a big problem)						
Somewhat problem		0.93(1.74)	0.02			[-2.49, 4.34]
No problem		4.82*(1.99)	0.11*			[0.91, 8.73]
Government assistance (no)						
Yes		1.23(1.85)	0.03			[-2.40, 4.86]
Constant		119.35***(19.71)				[80.65, 158.05]

Note. OLS = ordinary least squares; reference categories are indicated in parentheses. *B* = unstandardized coefficient, SE = standard error, *b* = standardized coefficient, CI = 95% confidence interval. * *p* < 0.05, *** *p* < 0.001

## Results for math problem skills

The effect size of this model is small (Cohen’s f² = 0.06). Table 4 presents the association between WJ-AP and various variables. First, we found that household income was associated withchildren’s WJ-AP scores: the higher the household income, the higher the WJ-AP (*B* = 6.67, *p* < 0.05). Second, in school neighborhoods, schools with no problems showed higher WJ-AP scores compared to schools with a big crime problem (*B* = 4.82, *p* < 0.01). According to the table, the strongest negative predictor was the school neighborhood with no problems (*b* = 0.11, *p* < 0.01).

**Table 5:** OLS results for Digit Span

Variables		B(SE)	b			95% CI
**Individual Level**						
Sex (boy)						
girl		0.42(0.22)	0.07			[-0.02, 0.84]
Race/Ethnicity (White)						
African American		-0.38 (0.37)	-0.06			[-1.10, 0.34]
Hispanic		-0.34(0.40)	-0.05			[-1.12, 0.45]
Other		-0.62(0.67)	-0.04			[-1.94, 0.70]
		-0.01(0.02)	-0.02			[-0.05, 0.03]
**Family Level**						
Mother’s age						
Household income		4.51(0.00)	0.00			[0.00, 0.00]
Mother’s marital status (married)						
separated		-0.06(1.00)	-0.00			[-2.02, 1.90]
divorced		-1.94(1.76)	-0.04			[-5.39, 1.51]
cohabiting		0.35(0.32)	0.06			[-0.27, 0.97]
Romantic not living together		-0.00(0.34)	-0.00			[-0.68, 0.67]
Not romantic		0.37(0.44)	0.04			[-0.27, 1.00]
Mother’s depression (no)						
Yes		-0.40(0.27)	0.06			[-0.93, 0.13]
Mother’s educational level (less than high school)						
High school or equivalent		0.38(0.32)	0.05			[-0.25, 1.01]
Some college teaching		0.40(0.28)	0.80			[-0.07, 1.04]
College or graduate		1.20*(0.49)	0.10*			[0.25, 2.15]
**Community/Environmental Level**						
School type (public)						
School with a magnet program		-0.05(0.60)	-0.00			[-1.22, 1.12]
School of choice		-0.11(0.38)	-0.01			[-0.85, 0.62]
School environment with crime (a big problem)						
Somewhat problem		-0.30(0.26)	-0.05			[-0.82, 0.22]
No problem		0.41(0.30)	0.06			[-0.18, 1.00]
Government assistance (no)						
Yes		0.40(0.28)	0.06			[-0.15, 0.95]
Constant		10.31***(3.00)				[4.45, 16.18]

Note. OLS = ordinary least squares; reference categories are indicated in parentheses. *B* = unstandardized coefficient, SE = standard error, *b* = standardized coefficient, CI = 95% confidence interval. * *p* < 0.05, *** *p* < 0.001

### Results for digit span

The effect size of this model is small (Cohen’s f² = 0.04). Table 5 presents OLS regression estimates predicting Digit Span. We found that children whose mothers had college or graduate degrees exhibited a higher Digit Span than children whose mothers had less than a complete high school education (*B* = 1.20, *p* < 0.05). Also, this variable was the strongest and the most positive predictor in the regression model (*b* = 0.10, *p* < 0.05).

**Table 6:** OLS results for Woodcock-Johnson passage comprehension (WJ-PC)

Variables		B(SE)	b			95% CI
**Individual Level**						
Sex (boy)						
girl		2.75*(1.22)	0.08*			[0.36, 5.14]
Race (White)						
African American		-1.23(2.07)	-0.04			[-5.37, 2.77]
Hispanic		-1.32(2.24)	-0.03			[-5.73, 3.08]
Other		-1.04(3.80)	-0.01			[-8.50, 6.43]
**Family Level**						
Mother’s age		-0.13(0.12)	-0.04			[-0.37, 0.11]
Household income		2.72(0.00)	0.04			[0.00, 0.00]
Mother’s marital status (married)						
separated		-5.68(5.63)	-0.04			[-16.73, 5.38]
divorced		-7.20(9.92)	-0.03			[-26.68, 12.28]
cohabiting		-0.14(1.78)	-0.00			[-3.64, 3.36]
Romantic not living together		-2.14(1.94)	-0.06			[-5.95, 1.66]
Not romantic		-1.25(2.50)	-0.02			[-6.36, 6.82]
Mother’s depression (no)						
Yes		-1.42(1.52)	-0.03			[-4.36, 1.61]
Mother’s educational level (less than high school)						
High school or equivalent		0.42(1.80)	0.01			[-3.12, 3.96]
Some college teaching		2.26(1.60)	0.07			[-0.89, 5.40]
College or graduate		5.90*(2.73)	0.09*			[0.54, 11.26]
**Communiy/Environmental Level**						
School type (public)						
School with a magnet program		0.23(3.36)	0.00			[-6.34, 6.822]
School of choice		3.75(2.12)	0.06			[-0.40, 7.90]
School neighborhood with crime (a big problem)						
Somewhat problem		1.34(1.49)	0.04			[-1.58, 4.26]
No problem		4.42**(1.71)	0.12**			[1.07, 7.77]
Government assistance (no)						
Yes		-1.42(1.52)	-0.3			[-4.52, 1.68]
Constant		98.05***(16.86)				[64.95, 131.15]

Note. OLS = ordinary least squares; reference categories are indicated in parentheses. *B* = unstandardized coefficient, SE = standard error, *b* = standardized coefficient, CI = 95% confidence interval. * *p* < 0.05, ** *p* < 0.01, *** *p* < 0.001

### Results for reading comprehension

The effect size of this model is small (Cohen’s f² = 0.05). Table 6 presents OLS regression estimates predicting WJ-PC. We found that sex was associated with WJ-PC. Girls exhibited higher WJ-PC scores than boys (*B* = 2.75, *p* < 0.05). Next, school neighborhoods without problems showed higher WJ-PC scores than school neighborhoods with a large crime problem (*B* = 4.42, *p* < 0.01). Finally, regarding mothers’ educational levels, mothers with either college or graduate degrees showed higher WJ-PC than mothers with less than high school degrees (*B* = 5.90, *p* < 0.05). According to the table, the strongest positive predictor was the mother’s educational level, either college or graduate level (*b* = 0.09, *p* < 0.05).

### Summary of findings

First, the school neighborhood factor was associated with three cognitive assessments: PPVT-III, WJ-AP, and WJ-PC. In other words, children in school neighborhoods without problems were reported to have higher scores in verbal intelligence, reading comprehension, and math compared to children in school neighborhoods with a big problem. Second, mothers’ educational levels were significantly related to three cognitive assessments, PPVT-III, Digit Span, and WJ-PC: children whose mothers had college or graduate degrees showed higher scores in the three areas than children whose mothers had less than complete high school educations. Third, children’s race was correlated with PPVT-III scores: African American and Hispanic children exhibited lower PPVT-III scores compared to White children. In addition, children whose mothers identified as being in a romantic relationship but not livingwith their partner also showed lower PPVT-III scores than children whose mothers were married. Other than verbal skills, race/ethnicity and marital status did not show a significant relationship with other cognitive areas. Household income was significantly associated with WJ-AP: children from higher-income families exhibited higher math skills than children from lower-income households. In addition to the math skills, household income did not show a significant association with other cognitive assessments. Among all assessments, only WJ-PC showed a correlation with sex: girls reported higher WJ-PC scores than boys. Overall, the PPVT-III assessing verbal skills was correlated with various social-ecological factors, such as race/ethnicity, mother’s marital status, mother’s educational level, and school neighborhood; accordingly, the WJ-PC assessing reading comprehension skills was also related to several social-ecological factors, such as sex, school neighborhood, and mother’s educational level.

## Discussion

Our study demonstrated that children’s cognitive development is influenced by a variety of family and social factors. We discovered that the factors showing the most significant associations with children’s cognitive development were the school neighborhood and the mother’s educational level. First of all, our study’s findings on the association between school neighborhoods and children’s cognitive development are consistent with previous studies [12, 16, 31]. Schools in poverty are likely to have lower levels of quality education and a lack of resources, and their environment is likely to be worse than those of schools in affluent neighborhoods. These situations have a negative influence on children’s developmental areas. These findings also highlight the importance of community and school environments on elementary school-aged children’s cognitive development.

Secondly, concerning family dynamics, the educational level and family structure (marital status) significantly impacted children’s cognitive outcomes in this study. As indicated by Harding et al. (2015), maternal education could be an essential tool that helps children experience other educational resources, such as being exposed to their mothers’ educated friends [13]. Furthermore, our study outcome associated with mothers’ marital status confirms that the family structure is vital for children’s cognitive/academic development (particularly verbal skills) [15]. Prior studies have primarily focused on the challenges faced by children from divorced or separated families when examining the impact of family dynamics on child development [40]. However, our research has identified a more intricate and specialized modern family setup that includes non-marital relationships, such as romantic partnerships where the individuals do not live together. This broader perspective could offer valuable insights into how the evolving family dynamics of today might affect a child’s cognitive development. As discussed by Fagan (2011), married mothers tend to have stronger reading and writing skills and higher household incomes, which could influence their children’s literacy levels [15]. Our study also supported the previous study by Lee and McLanahan (2015) that a mother’s marital status was significantly associated with PPVT scores. These research findings have shed light on the significance of a mother’s marital status as a protective factor in the connection between family structure and children’s cognitive growth.

Moreover, our research results regarding the link between household income and children’s math skills highlighted the work of Alves and colleagues [14]. They also noted that the ‘school’ variable did not significantly impact children’s cognitive abilities, while family circumstances were found to be a more influential factor in cognitive performance. This aligns with our findings, which indicated no significant relationship between the type of school and children’s cognitive development. We suspected that family and school environmental factors had a stronger correlation with children’s cognitive growth than the type of school attended. Consequently, this suggests that family structure, particularly mothers’ higher education and socioeconomic status, is closely associated with children’s cognitive success.

In terms of individual level variables, African American and Hispanic children showed lower results in verbal abilities. When taking into account racial and ethnic influences, it is suggested that this notable outcome may be more pronounced when combined with other factors. For example, in the studies mentioned, disparities in verbal skills based on race could be more prominent when influenced by family factors (such as parental education level) and community factors (such as living in disadvantaged neighborhoods or experiencing racism) [8]. 

### Implications

Investigating factors associated with elementary school students’ academic achievement is crucial for identifying groups that may require additional support to improve learning outcomes. To meet the diverse needs of students, professional development training should be offered to teachers to enhance their understanding of culturally responsive teaching and better serve students from different sociocultural and linguistic backgrounds [35, 41]. According to our research, students attending school in a neighborhood with high levels of crime are more likely to face challenges in their cognitive process than children from school neighborhoods with no problems. Therefore, educators, parents, and families must have the necessary tools and strategies to support these students academically [42]. To achieve this, offering assistive technology or adaptive devices can help these students enhance their learning experience. Furthermore, the literature review suggests that encouraging parents to assist their children with schoolwork at home using educational apps can be advantageous [28]. 

While not the primary focus of our study, the FFCW dataset included questions regarding whether English was considered a second language, both in general and specifically within the classroom. Out of the children surveyed, 88 indicated that English was their second language, while the remaining children (*n* = 1634) stated otherwise. The use of English as a second language could potentially impact children’s cognitive abilities. It may not accurately represent the cognitive skills of racial and ethnic minority children when assessments are conducted in English, as evidenced in the FFCW dataset. Therefore, future research should consider evaluating cognitive outcomes using the children’s native language (e.g., Spanish).

Establishing a positive and encouraging learning atmosphere is essential for all students, particularly those facing challenges like low-income households, unstable family dynamics, and schools with high levels of crime, as highlighted in our research. Collaborating with all stakeholders, including school staff and parents, is essential for fostering such an environment. Children in school neighborhoods without crime problems were reported to have higher scores in verbal intelligence, reading comprehension, and math problems than children in school neighborhoods with high crime rates. Such findings consistently demonstrate the need to improve neighborhood factors, which requires collaboration among schools, community members, and local government. Interventions that can reduce neighborhood crime can be considered. Long-term voluntary organizations have been found to exhibit crime-reducing behaviors in neighborhoods [43]. For instance, Neighborhood Watch [44] “a group of people living in the same area who want to make their neighborhood safer by working together and in conjunction with local law enforcement to reduce crime and improve their quality of life” (para. 1) is effective in reducing neighborhood crime. Community policing aims to reduce and resolve issues related to crime and disorder in the community by involving law enforcement agencies and community members. The introduction of a neighborhood watch model has been shown to reduce the fear of crime.

Furthermore, mothers with relatively lower education levels may particularly benefit from parent education programs that provide essential skills and resources to support their children’s academic learning. In the long term, increasing parental education levels, especially for those who were disadvantaged youths who had to discontinue their education early and were unable to pursue higher degrees, can lead to improved learning outcomes for their children [45]. Parental educational attainment, as a significant socioeconomic indicator, has been found to positively influence various aspects of parenting behavior and child outcomes [24]. For example, parental education level can positively impact parent-child interactions and the structuring of the home environment to promote academic achievement [24]. Additionally, parental educational attainment can influence parental expectations for their children, which, in turn, may impact their academic achievements. In the short term, targeted parent education programs can effectively improve parenting behaviors, providing hands-on parenting skills to support children’s academic learning [42]. Such programs can empower parents with the knowledge and tools they need to actively engage in their children’s education and create a conducive learning environment at home.

At the school level, promoting parental involvement becomes exceptionally crucial for parents with low educational levels, as they are often from more disadvantaged schools and communities. In comparison to more advantaged parents, these parents may require additional information and support to effectively promote academic achievement for their children. For policymakers, understanding the unique barriers and needs of each group is essential to establishing effective parent involvement initiatives [33, 46]. Parent involvement can be beneficial for children from diverse family structures, including different marital statuses. The current study established associations between parent involvement and cognitive assessments for various demographic groups, including race and gender.

Overall, it is imperative to consider diversity, equity, and inclusion (DEI) principles in education to ensure that all sub-demographic groups have equal access to educational opportunities, to address existing achievement gaps [47] as well, and to foster culturally responsive educational environments. By fostering a more inclusive, supportive, and culturally responsive educational environment, we can work toward narrowing the disparities in academic outcomes and promoting equitable educational experiences for all students and teachers. Encouraging this environment also helps to show respect for students’ cultures, languages, and life experiences and to create a multicultural climate in the classroom by integrating multicultural awareness into the curriculum, school events, and activities [48]. Furthermore, educators should motivate students to explore diverse cultures and histories globally. This thorough examination can boost the self-esteem and pride of students from minority or immigrant backgrounds. Lastly, in DEI education, teachers should avoid the use of racial and ethnic stereotypes and recognize the multiple perspectives that exist in the world. This approach helps students appreciate unique identities within a positive learning environment [48]. 

Although this study showed significant effects of social-ecological factors on children’s cognitive areas, it had some limitations. First, this study used a secondary cross-sectional dataset. Therefore, it is difficult to observe how those social-ecological variables affect children’s cognitive outcomes. Second, FFCW is collected from major metropolitan areas in the U.S. Therefore, children excluded from this dataset would have different outcomes than those we sampled from the data. As a result, any studies that use these study results should be cautious regarding generalizability. In the same vein, these data were collected in the U.S.; hence, when applying this study outcome, researchers or educators in other countries (e.g., East Asia, Europe) need to consider their nations’ educational policies, systems, and children’s environments.

Lastly, speaking of the magnitude of the study, each model had a small effect size, which implied that independent variables had a small effect on dependent variables. However, the most effective way to understand Cohen’s *d* is to compare it with other effects found in the literature and to recognize its role in illustrating practical differences, which can help summarize the statistically significant differences observed in the study’s findings [37]. 

## Conclusion

Our main focus was to identify the most predictable variables that were strongly linked to different cognitive areas, such as the impact of a mother’s college or graduate degree on verbal skills, digit span, and reading comprehension. This information could help in finding ways to support elementary school children in areas of need by reaching out to mothers with lower levels of education. This could involve providing educational resources to help children improve in these areas at home as well as educating parents about child development in both the community and school settings. Furthermore, through a thorough assessment of various cognitive domains using the social-ecological framework, we can determine the particular levels that need to be targeted to enhance specific cognitive areas in children. For instance, to enhance children’s reading comprehension, it is vital to focus more on boys, children in schools facing challenges, and children whose mothers have lower levels of education.

Overall, development is multidimensional and dynamic. According to our study findings, elementary school children are significantly influenced by various contexts, particularly family and community settings. In particular, our study addressed the importance of race/ethnicity, the mother’s educational level, family structure, household income, and the school neighborhood in the relationship with the specific area of elementary school students’ cognitive areas. Although this study has noticeable limitations, its findings will build upon the expertise and knowledge of middle childhood educators, policymakers, and researchers who should support children and their families by providing effective educational resources and opportunities for positive nurturing and mentoring within the school and community.

## Data Availability

The data, FFCWS is publicly available.

## Footnotes

1 Cohen (1988) categorized effect size as small when the f-square is > = 0.02; medium when the f-square is > = 0.15; and large when the f-square is > = 0.35 [38].
